# Intelligence

**Published:** 1832-03

**Authors:** 


### INTELLIGENCE.

### *History of the Progress of the* Malignant Cholera *in England and Scotland.*

Under the above title an admirable article was given in The Lancet of February 4th, of which the following is an abstract:

Importation into England. Upon this subject there has been much contradictory evidence; but it appears that the *importation* into Sunderland of the *blue cholera* has not as yet been *proved*, but that it is rendered highly probable. Where is there, in the whole history of disease, an example of a malady travelling, *without human aid*, across a wide tract of sea, selecting one particular port for its entrance into a certain country, confining its early influence to a circle of ground not one hundred yards in diameter, and then creeping on slowly in various directions? The occurrence is unprecedented in the chronology of disease, and impossible according to the recognised physical attributes of the air we breathe, and of the terrestrial surfaces which we inhabit.

Progress in Sunderland. It appears from various publications, that, during the entire Sunderland epidemic, the total amount of seizures and mortality up to the 26th January was 536 cases and 202 deaths. Of the medical topography of the town no good account has yet been published: its population is about 40,000. During the epidemic, the maximum number of cases did not exceed thirty daily, and that for a very limited period. The malady attacked comparatively few individuals in comfortable circumstances, and was most fatal among females, old, or very young persons, and those previously infirm or of intemperate habits. No meteorological phenomenon of the least importance marked the prevalence of the cholera in Sunderland, or characterised its subsequent extension.

About the second week in December, the villages around Sunderland began to be affected. In Newcastle, as in Sunderland, the disease was at first confined to the banks of the river: Sandgate street, a long and narrow winding street along the left bank of the Tyne, was, with the lanes running from it, chiefly infected. The population of Newcastle is estimated at 70,000: the total of cases up to the 30th of January, 888; deaths, 284. The same class of persons were attacked as in Sunderland; and, up to the 25th December, the usual rate of seizures had been from one to fifteen daily.

At this time Gateshead was visited by a severe irruption of the disease, as appears from the following extract from Dr. Adair Lawrie's Essay:

"Gateshead lies on the right bank of the Tyne, opposite to Newcastle, with which it is connected by a bridge. The population of the town of Gateshead is probably 10,000, and of the whole parish 15,000. The communication between the towns is necessarily very great. Up to the 25th December, only two cases of cholera had occurred at Gateshead, although the disease had prevailed at Newcastle for eighteen days. On the evening of the 25th December cases began to be frequent, and by ten o'clock on Monday morning, forty had been seized, and ten had died. At ten o'clock on the 27th (Tuesday), there had been ninety-nine cases and forty-two deaths from the Sunday evening. I visited patients in Gateshead-fell, three miles from the town, on Tuesday morning. In one small village there had been ten cases and five deaths."

In Gateshead, up to the 30th January, 391 persons had been attacked, of whom 141 died. Other villages around Gateshead and Newcastle, especially to the north, now suffered severely.

Scotland. Forsaking now the continuous routes and creeping pace which had characterised its progress during the early weeks of its existence in this country, the disease bounded over an interval of nearly one hundred miles, and appeared suddenly in Scotland, in the town of Haddington, situated on the North Tyne, sixteen miles south of Edinburgh: the population of this place is

5000. For the first ten days there were but fifty-six cases: on the 28th and 29th a sudden exacerbation of the epidemic took place, and on the latter day several very respectable persons were attacked, and perished. The latest accounts up to the time when the article in the Lancet was written, gave the cases at Haddington as seventy-five, and the deaths as forty-one.

The village of Tranent was next attacked, and, from the accounts to the 2d February, it appears there had here occurred 106 cases, and forty-one deaths.

On the 20th of January the disease shewed itself at Preston Pans, a parish contiguous to Tranent. The first case was that of a collier, aged thirty; the second, his child, aged two. The following day the coffinmaker, who made the coffins for Nos. 1 and 2, was attacked; as were also two children of a widow who assisted the family of the first cases during their distress.

According to the official reports, the first case of cholera occurred at Musselburgh, which is distant about three miles from Edinburgh, on Wednesday the 18th of January. Nine cases broke out the next day, three of which died in the course of the day. Next day, at three P.M., seventeen fresh cases were reported, and six deaths. From this time the disease continued to rage with a severity only paralleled in this country by the irruption at Gateshead, and, by the reports up to the 2d February, had attained the frightful height of 219 cases and ninety-three deaths, out of a population of 1500 persons: so rapid was the mortality, that a pit was necessarily dug for the general reception of the dead, and the gravedigger became one of its earliest tenants.

At the time the account was written, but few cases had occurred at Edinburgh. The authorities had issued directions for the closure of the theatres, and suspension of the evening service in the churches. The High School was closed, and Heriot's and Watson's Hospitals were stated, in a private letter, to have been placed in a state of seclusion, and the University shut up for a time. The bedding and apparel of the first patients were destroyed, and the *families removed*, WITH THEIR OWN CONSENT, to Queensbury house, a spacious building.

MODE OF EXTENSION. We regret that our limits do not permit us to quote without abridgment the clear and impartial statement in which the writer in the Lancet considers this most important question. He shews, by the clearest facts, that the disease *can* be communicated by human intercourse; and he further states that "the medical men, with few exceptions, (all, indeed, with whom we have met,) suffered from a symptom which is now known to be preliminary to this disease in a vast majority of cases, but which is perfectly amenable to therapeutic measures: we mean the diarrhœa, which, of course, attracted the immediate notice of the gentlemen alluded to, and was arrested by timely attention."

SYMPTOMS OF THE CHOLERA, *as it has appeared in England.* The great and important addition to our knowledge in this respect relates to the recognition of the preliminary diarrhœa, with which at least seven eighths of the cases in most of the districts have set in.* An ample collection of facts illustrative of this point have recently been published by the Central Board of Health, in the 2d number of the Cholera Gazette.

The following condensed extract is given from a report upon this subject by Dr. MACANN, who is at present superintending the districts of Northumberland and Durham:

"With reference to the questions which have been lately addressed to me from the Central Board, on the subject of diarrhœa as connected with cholera, I have now to state, that my observations and inquiries, in this part of the country, Sunderland, Newcastle, and Gateshead, all concur in leading me to adopt the following conclusions on that subject.

"1st. That the appearance or invasion of the proper choleric symptoms, viz.

* Mr. ORTON and other writers mention the very frequent occurrence of simple diarrhœa as a premonitory symptom of Indian cholera.—EDITOR.

the vomiting and purging of fluids, neither feculent nor bilious, with cramps and prostration, was preceded, in a great majority of the cases which have hitherto occurred, by a marked relaxation of the bowels, that is, by frequent fluid dejections, constituting the complaint denominated simple diarrhœa.

"2d. That this diarrhœa, in a great majority of cases, presented for a time no peculiar character, so that no man could tell, when called early to a patient labouring under it, whether an attack of cholera was impending or not.

"3d. That in many cases, however, the dejections were, from an early period, more analagous to those which take place in cholera than in diarrhœa, that is, were more fluid, whiter, and less feculent than they usually are in the latter complaint; and that in many of the other cases they assumed more or less of that character before the invasion or appearance of the proper choleric symptoms already referred to.

"4th. That cases of disease (as in paragraph 2d) have been met with in all classes of society, and been successfully treated as simple diarrhœa, by the ordinary remedies, viz. mild laxatives, astringents, opium, &c.

"5th. From the preceding observations it would appear, that in this country, hitherto, an attack of cholera has, generally speaking, been preceded by a relaxed state of the bowels; and that the dejections connected therewith have, sooner or later, presented to the careful observer some indications of the danger to which the patient was exposed.

"6th. For this reason, an early and assiduous attention to bowel complaints of all kinds, even the most trifling, must, as a measure of precaution and safety, be of the highest importance to all persons residing in our near infected districts."

In elucidation of the blue or collapse stage, a plate is given in the same number of the Lancet, which was taken from a drawing of a girl who died in Sunderland, after a few hours' illness.

In this country, it is said that not more than a dozen of dissections of cholera cases have taken place, and in these no appearances worthy of note have been detected. It is added, however, that the pathology of the malady has been investigated by another, and perhaps a more conclusive method; numerous analyses of the blood and dejected matters having been performed, in Newcastle, by Dr. O'Shaughnessy, and of the blood in Sunderland, by Dr. Clanny. The results obtained by each experimentalist coincide in denoting an immense loss of water and deficiency of saline matter in the blood; while Dr. O'Shaughnessy has detected the materials which are absent in the blood, in the peculiar dejected fluids. He has laid before the Central Board a voluminous report of his experiments, which is not yet published. Neither the experimental results nor the pathological inferences of Dr. Clanny are admitted to be correct by the writer in the Lancet. He considers it obvious, as far as the preceding statements entitle us to reason, that we must refer many of the symptoms of the malady to mechanical hyperæmia, or an inspissation of the blood, thus rendered too thick for pulmonary circulation. "That the leading symptoms may thus receive a satisfactory explanation, cannot be questioned; and we therefore wait with anxiety the publication in detail of the recent inquiries, and their further prosecution by those professional gentlemen now resident in the theatre of the epidemic, to whom the department of the chemical pathology of cholera has been confided by the Edinburgh Board of Health."

Treatment. The frequent preliminary symptom, diarrhœa, should be relieved as speedily as possible: by attention to this point many lives have been saved, and it is certain that the future amount of mortality may be materially diminished, if it be constantly kept in view. In the second, or collapse stage, mustard emetics are said to have been sometimes serviceable, when timely administered. This practice was first recommended by Dr. Gibson at Sunderland, and put into operation by Dr. Lindsey. The immediate restoration of the pulse has, in many cases, followed the action of this remedy, and the patient has passed through an apparently hopeless cold stage. The Central Board of Health has officially recommended this emetic, in doses of three teaspoonsful of mustard to half a pint of warm water. In children and very young persons, and individuals so paralysed by the disease that the powers of deglutition are impaired, some caution should be observed in its administration. The author of the article in the Lancet offers this suggestion in consequence of having been informed that, in more than one case, such severe spasms of the glottis, and irritation of the muscles of the larynx, were induced by the acrid powder, that the patients nearly perished of instant suffocation.* The inhalation of oxygen gas, and its injection into the rectum, were tried at Sunderland, without benefit. In Newcastle, Mr. Baird is said to have used a tobacco injection in six cases, with great advantage; but in another case thus treated by Dr. Lawrie, almost instant death supervened in the experiment. The next practical improvement in the treatment is thought of considerable importance, especially when viewed in connexion with the recent chemical researches which have been already noticed: the employment of copious injections of diluent fluids into the rectum is alluded to; a measure first practised by Dr. Gibson in one of the cholera hospitals at Newcastle, and since repeated with the most encouraging results.

The article concludes with some good remarks on "Sanatory Measures, and on the Benefit of Coast Quarantine."

---

### CHOLERA IN LONDON.

Does the "malignant cholera" really exist in London, or does it not? is a question that has, within the last three weeks, been discussed with the utmost vehemence. We lament to say, that facts and weight of authority are on the affirmative side; while the negative is supported by abundance of speculative doctrines, and by persons who have comparatively little claim to our confidence. That the malady which now exists in some parts of the metropolis is identically the same with the cholera that has prevailed in India and Europe, is positively affirmed by the practitioners who have witnessed the disease in both these parts of the world. The few medical persons who are opposed to this opinion have not seen the malignant cholera in other countries, and therefore they are not justified in assuming that tone of confidence with which they have ventured to address the public, through the medium of the daily papers. We leave entirely out of the question the lucubrations of the editors of some of the newspapers, as we take it for granted that medical men will not be influenced by such arrogant yet incompetent judges. Their only object appears to be to prevent mercantile men from suffering the least temporary inconvenience, even at the risk of the dissemination of a fatal pestilence. It is universally admitted that a malignant and rapidly fatal disease does exist at the present moment in London; but some deny it to be the Asiatic cholera, and affirm that it is a "fever" of a peculiar type; while a few even regard it as a modification of the common cholera of this country. If it be admitted that the disease is as much like "congestive fever" as spasmodic cholera, still the precautions that have been enforced by the government of the country are necessary; for the origin and progress of the disease amongst us have been such as to justify the strongest suspicion, even if not to afford positive proof, that it is communicable from person to person.

Against the opinion which maintains that this disease is nothing more than the common cholera of the country, we appeal to the experience of the oldest metropolitan practitioners, to which we may add our own conviction, which

* It is stated in the Report of the London Medical Society, (Lancet, Feb. 18th,) that the administration of a mustard emetic in one of the cases of cholera which has occurred in London, hastened the death of the patient. The medical attendants thought the woman would have died almost immediately after it was given. This was stated by Dr. Whiting.

results from a twenty years' pretty active observation of the diseases of London: during that time we have never seen but one fatal case of cholera, and, from all the information we can obtain from others, we have reason to believe that such an occurrence is very rare.

In order that our readers may have an opportunity of judging for themselves, we subjoin the detailed account, from the third number of the Cholera Gazette, of some of the cases which have recently attracted so much attention.

"CASE I.—Case of John James, æt. forty-eight, Ship scraper, working on board a Collier from Newcastle: died on Thursday, Feb. 9, at four o'clock in the morning, fourteen hours after leaving off work. Notes by Mr. JACKSON.

"On Wednesday, 8th February, I was desired to visit a sick man who was very ill, in Hanover street. It was about eight in the evening when I arrived; the following was the statement: That he had had a loose or irritable state of the bowels for some days past, but more particularly from the preceding Monday. He, however, followed his usual occupation till the day of attack, about two o'clock in the afternoon, (on board of a ship in the Regent or City Canal,) when he was obliged to relinquish it, from vomiting and cramps in his hands and feet. On my visit, he was laying on his left side, with his head and chest slightly bent forward, and the knees a little drawn up.

"The extremities, abdominal, lumbar, and pectoral muscles, were in a complete state of tonic spasm.

"The features of the face sunk and pinched, exhibiting an indescribable countenance of anxiety, suffering, and despair. The surface of the face blue, and peculiarly so the lips, as in a patient labouring under hydrothorax.

"The tongue cold and moist, with a white appearance as if he had been licking pipeclay. The breath nearly cold.

"Vomiting of a matter resembling turbid or muddy water, with thirst, and great pain at the pit of the stomach. Respiration greatly oppressed. The surface of the body cold, and the extremities most peculiarly so, giving the sensation of touching a dead body, covered with a clammy sweat. Of what kind or quantity the purging was, I did not ascertain.

"Not wishing to designate the case before me what I really thought it was, I called in Mr. Gaitskell, when we found the patient still worse in all the symptoms: the pulse gone; tongue cold, and also the breath; the breathing slow, and oppressed with a strong pain over the region of the heart, which prevented him from breathing. He was quite sensible throughout. His voice a whisper, with a curious thrill.

"We left him about eleven P.M., and he died about four next morning.

"DISSECTION of John James, ætat. forty-eight, about fifty hours after death.

"External appearances: The whole frame rigid, hard, and inflexible; fingers bent in and corrugated, of a light leaden colour, nails blue; the feet the same, but not in so obvious a proportion; the penis and testes were blue; the countenance natural, but the eyes much sunk. (The lividness of his countenance is reported by his relatives to have kept gradually disappearing since death.)

"Abdomen: The stomach distended with fluid, but the intestines moderately so, the latter presenting a slight blush; the jejunum, ileum, and colon were opened for a considerable distance in different places, and each contained a fluid resembling thin gruel; there was not the slightest appearance of bile or fæces, or odour of the latter, observable; the contents of the stomach were the same as those of the intestines, only rendered darker from the admixture of brandy, medicines, &c. The internal coats of the stomach and intestines were remarkably healthy.

"The bladder was contracted and firm, containing no urine, but a small quantity of fluid resembling that in the intestines. The pancreas and spleen healthy; the right kidney small, but the left one healthy and natural; the liver healthy, but, when cut into, the veins were loaded with black blood; the gall-bladder healthy, full of bile, and without gallstones.

"Thorax: The lungs natural, healthy, but much collapsed; no water in the chest or pericardium; the heart healthy, natural, large, with a considerable portion of fat on its superior surface. The right and left auricles were distended with blood, and I may here remark, that all the veins terminating in these cavities (as the venæ cavæ inferior and superior, and the pulmonary veins,) were the same. On removing the heart, and opening the auricles, the blood presented a thick, dark, tarry consistence, without intermixture of serum. The blood which flowed from the divided vessels had the same appearance. The ventricles were free from blood, but each contained a fibrinous coagulum, about the size of a nutmeg.

"The Head: On removing the calvarium, the brain presented nothing particular, only the venous circulation was turgescent with dark-coloured blood. The substance of the brain itself was beautifully firm and healthy, and the ventricles contained no fluid whatever; neither was there any effusion, or particular turgescence of the blood-vessels, on the base of the cerebellum and cranium.

"Throughout the examination of the various organs, there was not the least trace of any disease, either organic or constitutional, that could at all affect life, or even produce temporary inconvenience.

"John Jackson, Surgeon.

"*Rotherhithe; Feb.* 12, 1832." "W. Gaitskell, sen., Surgeon."

"*Details of three Fatal Cases at Limehouse.*

"CASE I. FATAL IN FOURTEEN HOURS.

"Mary Ferguson, residing at No. 2, White's rents, in the parish of St. Ann's, Limehouse, aged twenty-five, went to bed well on Saturday night last; was attacked, about four o'clock on Sunday morning, with purging and vomiting, attended with violent cramps of the legs and thighs, which continued till seven o'clock, when the extremities of the face assumed a blue colour. The dejections from the bowels ran off involuntarily, accompanied with extreme coldness, great prostration of strength, and contracted appearance of the countenance; which appearances continued to increase with the purging till half-past two o'clock, at which time application for medical attendance was first made.

"The tongue and breath were found to be rather colder than natural, and the dejections from the bowels to have the appearance of dirty gruel.

"She was removed to the workhouse, a distance of two hundred yards. Laudanum, brandy, and other stimulants were used, together with dry heat. She died a little after six o'clock the same evening.

"CASE II. FATAL IN FIFTEEN HOURS.

"Caroline Shea, of Walnut-tree court, St. Ann's, Limehouse, aged eleven years; was attacked about the same time and in the same manner as the above case, only the skin was not so dark: the same means were used. Medical assistance was called in ten hours from the commencement of the disease. She was removed at eleven o'clock at night, and died at seven this morning.

"CASE III. THREE DAYS' ILLNESS.

"Margaret Shea, aged forty, had been affected with a looseness for several days previous to Thursday morning last, when she was seized with constant vomiting and purging, accompanied with cramps of the limbs; the skin was cold; she had insatiable thirst; complained of general pain all over her, except griping of the bowels. Medical assistance was called on Friday noon: upon inquiry, she stated that she had passed no water. Blisters were ordered to the pit of the stomach; stimulants, laudanum, with other cordials, were used; she seemed to rally a little till Sunday forenoon, when she became very restless, and evidently losing strength; was removed to the workhouse, and died at one o'clock this morning. The child, Caroline Shea, was her daughter, and had been in attendance on her during her illness.

"Thos. W. Barnet, Surgeon, 75, Fann-street, Limehouse.

"*Feb.* 13*th.* "Wm. S. Cumming, Surgeon, Limehouse."

After perusing these cases, we think there are few practitioners who will deny that the disease here described is different from any with which the inhabitants of London have hitherto been attacked; and any doubt that may remain as to its identity with the malignant cholera will be removed by turning to the description of the latter disease as it has been given by the best authorities.

The following is the last report that has been published at the time we write.

*Daily Report of Cholera Cases. Central Board of Health, Whitehall.*

| Place. | Date. | Remaining a. lastReport. | New Cases. | Dead. | Recovered. | Remaining. | Total Cases from Commencement of Disease. | Total Deaths from Commencement of Disease. |
|---|---|---|---|---|---|---|---|---|
| Limehouse . | Feb. 25 | 1 | 0 | 1 | ... | 0 | 8 | 7 |
| Afloat in River . | | 3 | 3 | 2 | ... | 4 | 11 | 6 |
| Whitechapel . | | 0 | 2 | 2 | ... | 0 | 3 | 3 |
| Southwark . . | | 2 | 1 | 1 | ... | 2 | 29 | 16 |
| Lambeth . . . | | 1 | 0 | 0 | ... | 1 | 5 | 4 |
| Bermondsey . . | | 0 | 3 | 3 | ... | 0 | 3 | 3 |
| St. Pancras . . | | 0 | 2 | 1 | ... | 1 | 2 | |
| Rotherhithe . . | | 0 | 1 | 1 | ... | 0 | 4 | 4 |
| St. Giles's . . | | 0 | 3 | 2 | ... | 1 | 3 | 2 |
| | Total | 7 | 15 | 13 | ... | 9 | 68 | 46 |
| Cases before reported at St. Marylebone and Ratcliffe . . . | | | | | | | 2 | 2 |
| | | | | | | | 70 | 48 |

(*Signed*) W. Maclean, *Secretary.*

### METEOROLOGICAL JOURNAL,

By Messrs Harris and Co. Mathematical Instrument Makers, 50, High Holborn.

| | Moon. | Rain gauge. | Thermometer. | | | Barometer. | | De Luc's Hygrometer. | | Winds. | | Atmospheric Variation. | | |
|---|---|---|---|---|---|---|---|---|---|---|---|---|---|---|
| | | | 9a.m. | max. | min. | 9a.m. | 10p.m. | 10p.m. | 9a.m. | 9 a.m. | 10p.m. | 9 a.m. | 2 p.m. | 10 p.m. |
| Jan. 20 | | | 34 | 40 | 34 | 29.94 | 29.94 | 68 | 70 | S | SW | foggy | foggy | foggy |
| 21 | | | 40 | 42 | 38 | 30.03 | 30.18 | 70 | 70 | SW | SW | | | |
| 22 | | | 41 | 43 | 40 | .15 | .15 | 70 | 70 | SE | SE | fine | fine | |
| 23 | | | 42 | 43 | 36 | .13 | .16 | 70 | 70 | S | SSW | foggy | cloudy | fine |
| 24 | ☾ | | 42 | 44 | 34 | .05 | 29.87 | 70 | 71 | SSW | S | fine | fine | cloudy |
| 25 | | | 38 | 40 | 37 | 29.65 | .68 | 70 | 68 | SW | SW | rain | fine | foggy |
| 26 | | | 40 | 43 | 33 | .67 | .64 | 68 | 69 | WSW | SSW | foggy | cloudy | showery |
| 27 | | | 35 | 38 | 30 | .80 | .96 | 69 | 67 | NE | NNE | snow | cloudy | fine |
| 28 | | | 32 | 36 | 38 | 30.08 | 30.02 | 67 | 67 | N | WSW | fine | fine | cloudy |
| 29 | | | 43 | 45 | 36 | .23 | .18 | 67 | 68 | NW | NW | cloudy | showery | cloudy |
| 30 | | | 42 | 42 | 41 | .14 | .03 | 68 | 67 | W | W | cloudy | cloudy | foggy |
| 31 | | | 42 | 43 | 33 | 29.90 | .70 | 64 | 62 | WSW | SSW | foggy | cloudy | foggy |
| Feb. 1 | ● | | 36 | 47 | 38 | .25 | 29.11 | 62 | 65 | SSE | SSW | cloudy | fine | cloudy |
| 2 | | | 46 | 46 | 36 | .08 | .05 | 67 | 65 | SSW | SW | fine | | fine |
| 3 | | | 38 | 51 | 37 | .42 | .60 | 65 | 68 | WSW | SW | | | cloudy |
| 4 | | | 50 | 52 | 42 | .69 | .81 | 68 | 67 | SW | SW | showery | | |
| 5 | | | 48 | 53 | 47 | .83 | .85 | 67 | 65 | | SSW | cloudy | showery | fine |
| 6 | | | 47 | 51 | 40 | .62 | .50 | 65 | 65 | S | SSW | fine | rain | fine |
| 8 | | | 42 | 43 | 37 | .80 | 30.00 | 64 | 64 | WNW | NW | | fine | |
| 7 | | | 43 | 46 | 43 | 30.16 | .19 | 64 | 65 | SW | SW | foggy | | |
| 9 | ☽ | | 46 | 48 | 39 | .19 | .26 | 66 | 65 | SW | N | rain | cloudy | |
| 10 | | | 43 | 46 | 36 | .35 | .33 | 65 | 64 | N | N | foggy | fine | |
| 11 | | | 40 | 42 | 33 | .14 | .03 | 64 | 63 | NNE | NNE | fine | cloudy | |
| 12 | | | 36 | 42 | 35 | 29.96 | 29.91 | 64 | 66 | NE | ENE | | cloudy | sleet |
| 13 | | | 39 | 41 | 36 | .91 | .91 | 65 | 64 | ESE | ESE | foggy | cloudy | cloudy |
| 14 | | | 37 | 38 | 30 | .87 | .87 | 64 | 63 | E | SE | | cloudy | fine |
| 15 | | | 34 | 36 | 29 | .87 | .83 | 63 | 65 | ESE | NE | | | |
| 16 | ○ | | 33 | 39 | 34 | .65 | .54 | 65 | 65 | SE | SW | | fine | fine |
| 17 | | | 39 | 41 | 38 | .58 | .81 | 65 | 65 | SSW | NNE | rain | cloudy | cloudy |
| 18 | | | 41 | 43 | 35 | 30.02 | 30.12 | 65 | 64 | N | NNE | showery | fine | fine |
| 19 | | | 39 | 42 | 31 | .12 | .14 | 64 | 64 | E | E | fine | cloudy | |

The Rain gauge having been frozen, the quantity cannot be given.

